# The pancreatitis-associated protein VMP1, a key regulator of inducible autophagy, promotes Kras^G12D^-mediated pancreatic cancer initiation

**DOI:** 10.1038/cddis.2016.202

**Published:** 2016-07-14

**Authors:** C Loncle, M I Molejon, S Lac, J I Tellechea, G Lomberk, L Gramatica, M F Fernandez Zapico, N Dusetti, R Urrutia, J L Iovanna

**Affiliations:** 1 Centre de Recherche en Cancérologie de Marseille (CRCM), INSERM U1068, CNRS UMR 7258, Aix-Marseille Université and Institut Paoli-Calmettes, Parc Scientifique et Technologique de Luminy, Marseille, France; 2 Laboratory of Epigenetics and Chromatin Dynamics, Gastroenterology Research Unit, Departments of Biochemistry and Molecular Biology, Biophysics, and Medicine, Mayo Clinic, Rochester, USA; 3 Department of Surgery, University of Cordoba, Cordoba, Argentine; 4 Schulze Center for Novel Therapeutics, Mayo Clinic, Rochester, USA

## Abstract

Both clinical and experimental evidence have firmly established that chronic pancreatitis, in particular in the context of Kras oncogenic mutations, predisposes to pancreatic ductal adenocarcinoma (PDAC). However, the repertoire of molecular mediators of pancreatitis involved in Kras-mediated initiation of pancreatic carcinogenesis remains to be fully defined. In this study we demonstrate a novel role for vacuole membrane protein 1 (VMP1), a pancreatitis-associated protein critical for inducible autophagy, in the regulation of Kras-induced PDAC initiation. Using a newly developed genetically engineered model, we demonstrate that VMP1 increases the ability of Kras to give rise to preneoplastic lesions, pancreatic intraepithelial neoplasias (PanINs). This promoting effect of VMP1 on PanIN formation is due, at least in part, by an increase in cell proliferation combined with a decrease in apoptosis. Using chloroquine, an inhibitor of autophagy, we show that this drug antagonizes the effect of VMP1 on PanIN formation. Thus, we conclude that VMP1-mediated autophagy cooperate with Kras to promote PDAC initiation. These findings are of significant medical relevance, molecules targeting autophagy are currently being tested along chemotherapeutic agents to treat PDAC and other tumors in human trials.

Pancreatic ductal adenocarcinoma (PDAC) is the fourth leading cause of cancer-related deaths in the Western world and predicted to be the second one in 2030.^1^ The initiation, progression, and maintenance of PDAC results from the interplay of genetic events combined with other multiples less well-characterized factors.^2^ The genetic alterations contributing to PDAC pathogenesis have been extensively studied and clearly determined. Among the most common genetic alterations contributing to pancreatic carcinogenesis, oncogenic mutations in *KRAS* are the most frequently detected not only in frank PDAC but also in its best characterized preneoplastic disease, namely chronic pancreatitis. Oncogenic KRAS signals initiate acinar-to-ductal metaplasia, a step essential for the formation of preneoplastic lesions, that together with mutations in tumor suppressors such as *CDKN2A*, *TP53*, and *SMAD4* occurring during the progression from pre-neoplastic pancreatic intraepithelial neoplasia (PanIN) lesions, promotes the development of invasive cancer.^3^ Thus, oncogenic mutations in KRAS are necessary to initiate cancer formation and as such remain one of the most studied genetic alterations in PDAC. However, the entire repertoire of pathways contributing with this phenomenon remains elusive.

Autophagy has been proposed as a cellular contributing to pancreatic carcinogenesis and in particular the tumor initiating effects of KRAS.^4, 5, 6, 7^ Indeed, oncogenic KRAS generates a metabolic stress characterized by a temporary deficit in energy which must be compensated by increasing metabolic resources through the activation of autophagy.^4, 5, 6, 7^ However, the role of autophagy as pro- or anti-tumor is largely debated because it seems to be conditioned by the pathway regulating this phenomenon, the genomic status of the transforming cell population as well as both the physiological and pathological context in which this process is activated.^8, 9^ Consequently, more work is needed to define the repertoire of autophagy mediators, and pathways, which either promote or antagonize PDAC development. Thus, autophagy mediators, which also work in pancreatitis, are good candidates as modifiers of the effect of oncogenic pathways leading to pancreatic transformation.

We have previously identified a pancreatitis-induced transmembrane protein known as vacuole membrane protein 1 (VMP1), which regulates an inducible form of autophagy.^10, 11^ Mechanistically, VMP1 is involved in the phagophore formation by directly binding to beclin1.^11^ Noteworthy, VMP1 expression is transcriptional induced by oncogenic KRAS via a GLI3-p300-dependent mechanism.^12^ Therefore, VMP1 is strongly induced by two complementary PDAC-promoting pathways, namely, pancreatitis and activated KRAS, which further support the hypothesis that this protein may be necessary to initiate neoplastic transformation. To test this hypothesis, we developed a novel mice model in which the VMP1 is induced specifically in the pancreas by doxycycline together with activation of the oncogenic Kras^G12D^. This model allowed us to evaluate the effects of VMP1 on PDAC initiation as well as serve as a platform for preclinical trials, which can evaluate the role of autophagy inhibitors on PanIN development. The results of these experiments support the hypothesis stated above and unravel, for the first time, a new role for VMP1-mediated autophagy in the promotion of KRAS-mediated PDAC initiation. Moreover, through a preclinical trial that uses chloroquine to inhibit autophagy we demonstrate that the promoting effects of VMP1 on initiation can be reversed. Thus, combined, these results reinforce the idea that distinct pancreatitis-associated pathways, in particular those that regulate autophagy, have the ability to contribute to the process of pancreatic carcinogenesis. Lastly, our findings further underscore the potential utility of inhibiting autophagy to inhibit PDAC growth.

## Results

### Development of the Pdx1-cre-Kras^G12D^/VMP1DsRed mice, a novel genetically engineered mouse model for studying the role of inducible autophagy on PDAC initiation

**Figure 1:**
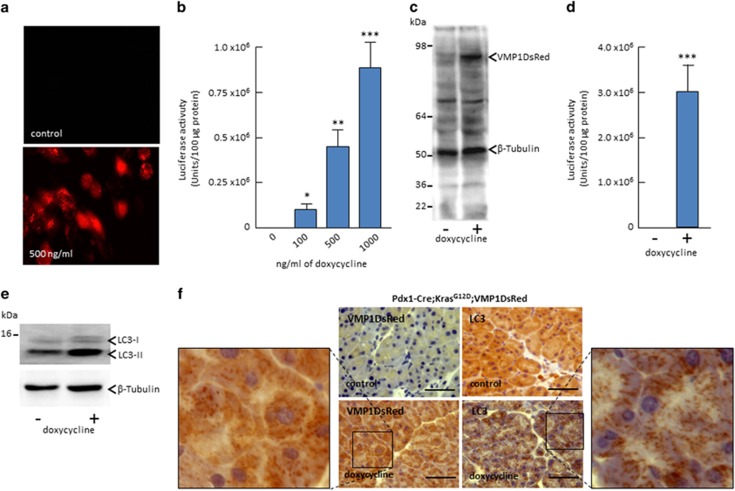
Construct Ptight-VMP1DsRed-IRES-Luciferase Renilla-SV40 polyA is functional *in vitro* and *in vivo*. VMP1DsRed-IRES-Luciferase Renilla construct was transfected in HEK293 cells and 24 h later treated with 500 ng/ml doxycycline and 24 h later DsRed expression was observed (**a**). Luciferase activity measured after 24 h of treatments with 100, 500, and 1000 ng/ml doxycycline (*P<0.05, **P>0.01, and ***P>0.001) mean±S.E.M (**b**). Expression of the VMP1DsRed (**c**) and Luciferase activity (**d**) in the pancreas of the transgenic mice treated or not with doxycycline dissolved in drinking water was measured by Western blot using a VMP1 antibody. Loading was controlled by using a *β*-Tubulin antibody. LC3 cleavage was measured by Western blot (**e**). Staining of VMP1dsRed and LC3 by IHC (**f**). VMP1dsRed and LC3-II isoform was more abundant in the pancreas of transgenic mice treated with doxycycline than in controls. IHC reveals punctate distribution in the pancreas of transgenic mice treated with doxycycline whereas it is not detected (VMP1dsRed) or homogeneously distributed in the cytoplasm (LC3) of controls animals. Scale bars=50 *μ*m

The VMP1 protein has recently elicited attention because it is a key regulator of autophagy in organisms ranging from *Caenorhabditis elegans* to mammals.^11, 13, 14^ As it regards to the pancreas, recent studies have demonstrated that when fused to green fluorescent protein, the transgenic expression of VMP1 in the exocrine cells of the murine gland, this protein increases the levels of autophagy.^11, 15^ In the current study, however, we sought to define the role of VMP1 in the regulation of PDAC initiation using a mechanistic design based on expressing VMP1 in murine pancreatic cells by creating a novel genetically engineered mouse model (GEMM) co-expressing VMP1-DsRed and oncogenic Kras^G12D^ in the exocrine pancreas. Because VMP1 is a regulator of ‘inducible autophagy' and this process can have either a positive or negative impact on the regulation of pancreatic cell growth depending upon the cellular context, we designed this GEMM to express a doxycycline inducible VMP1-DsRed protein from a bi-cistronic cassette that also contains an IRES-luciferase sequence (Figure 1). When crossed to Pdx1-Cre-‘Tet on' mouse, this animal allows us to control for the expression of the transgene by both fluorescence and luciferase assays. Indeed, Figure 1 shows that upon induction with doxycycline, these mice readily express both VMP1DsRed and luciferase. Notably, VMP1DsRed is induced as punctate in the pancreas of animals treated with doxycycline concomitantly with the relocalization of LC3 as dots (Figure 1). Thus, together, these results demonstrate that our novel genetically engineered mouse readily express VMP1 in an inducible and tractable manner (see Supplementary Figure 1), suggesting that it can become a useful tool for better understanding the role of this protein during PDAC initiation.

### Inducible expression of VMP1 promotes Kras^G12D^-mediated PDAC initiation

**Figure 2:**
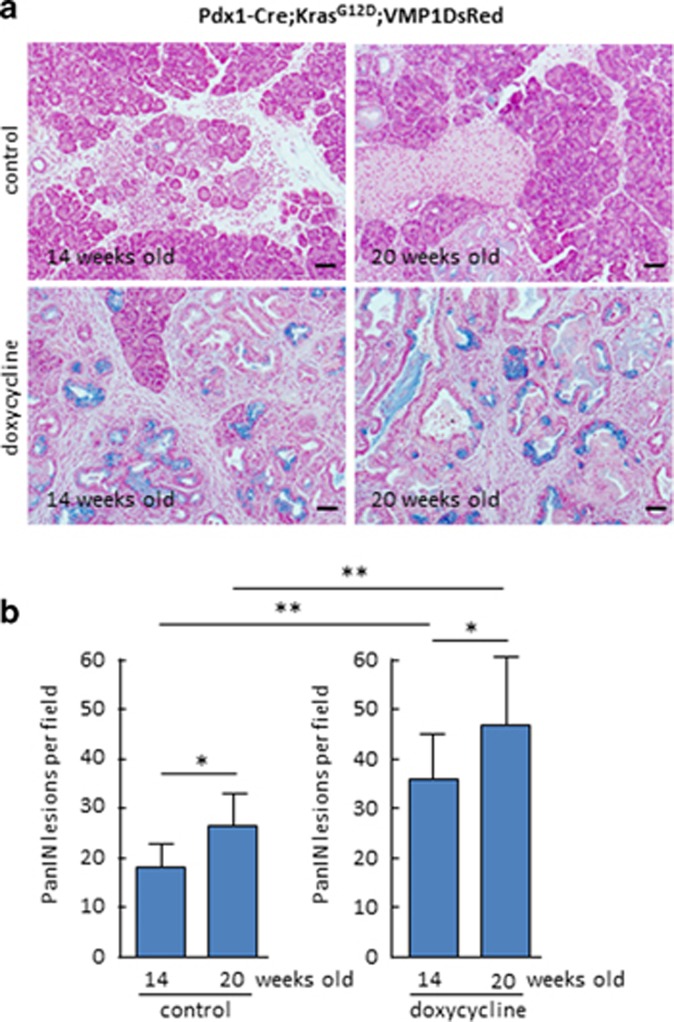
PanINs development is increased in Pdx1-cre;LSL-Kras^G12D^;VMP1DsRed mice. (**a**) Representative pictures of pancreas from Pdx1-cre;LSL-Kras^G12D^;VMP1DsRed mice stained with Alcian blue at 14 and 20 weeks old treated with doxycycline dissolved in drinking water. (**b**) Number of PanIN lesions per × 20 tissue field in Pdx1-cre; LSL-Kras^G12D^; VMP1DsRed treated with doxycycline or control at 14 and 20 weeks of age. (*P<0.05 and **P>0.01) mean±S.E.M. Scale bars=50 *μ*m

**Figure 3:**
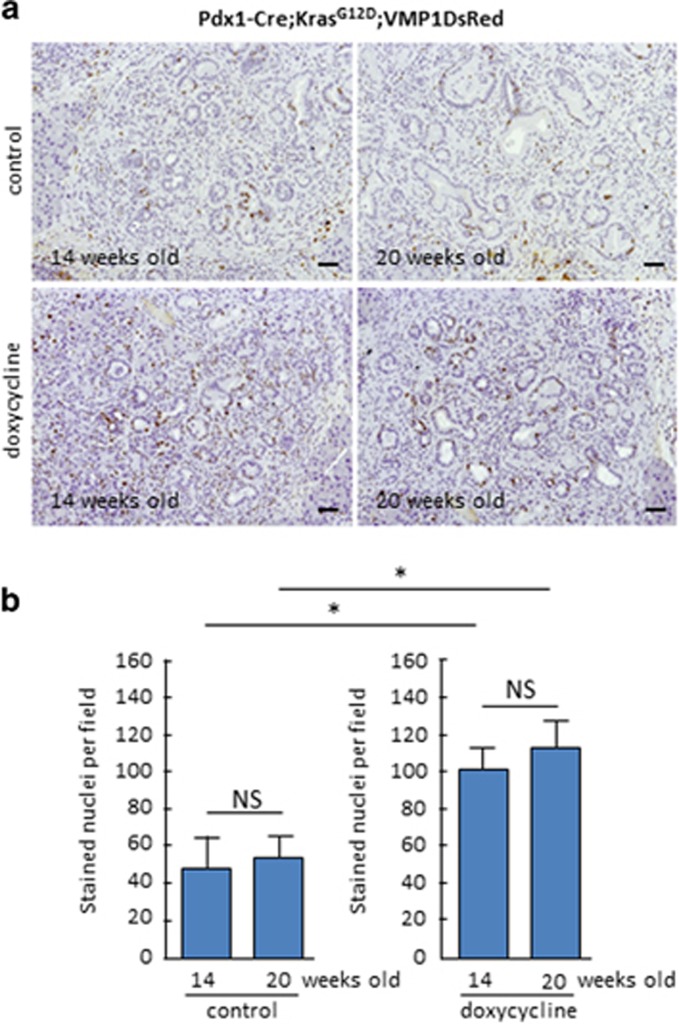
KI67 staining is increased in the pancreas of Pdx1-cre;LSL-Kras^G12D^;VMP1DsRed mice treated with doxycycline. Immunostaining of the Ki67 antigen was performed to estimate the proliferation activity in the pancreas of Pdx1-cre;LSL-Kras^G12D^;VMP1DsRed mice treated or not with doxycycline. (**a**) Representative pictures of pancreas from Pdx1-cre;LSL-Kras^G12D^;VMP1DsRed mice stained with anti-Ki-67 at 14 and 20 weeks old treated with doxycycline dissolved in drinking water. (**b**) Quantification of positive nuclei per × 20 tissue field in Pdx1-cre;LSL-Kras^G12D^;VMP1DsRed treated with doxycycline or control at 14 and 20 weeks of age. (*P<0.05 and NS corresponds to not significant) mean±S.E.M. Scale bars=50 *μ*m

**Figure 4:**
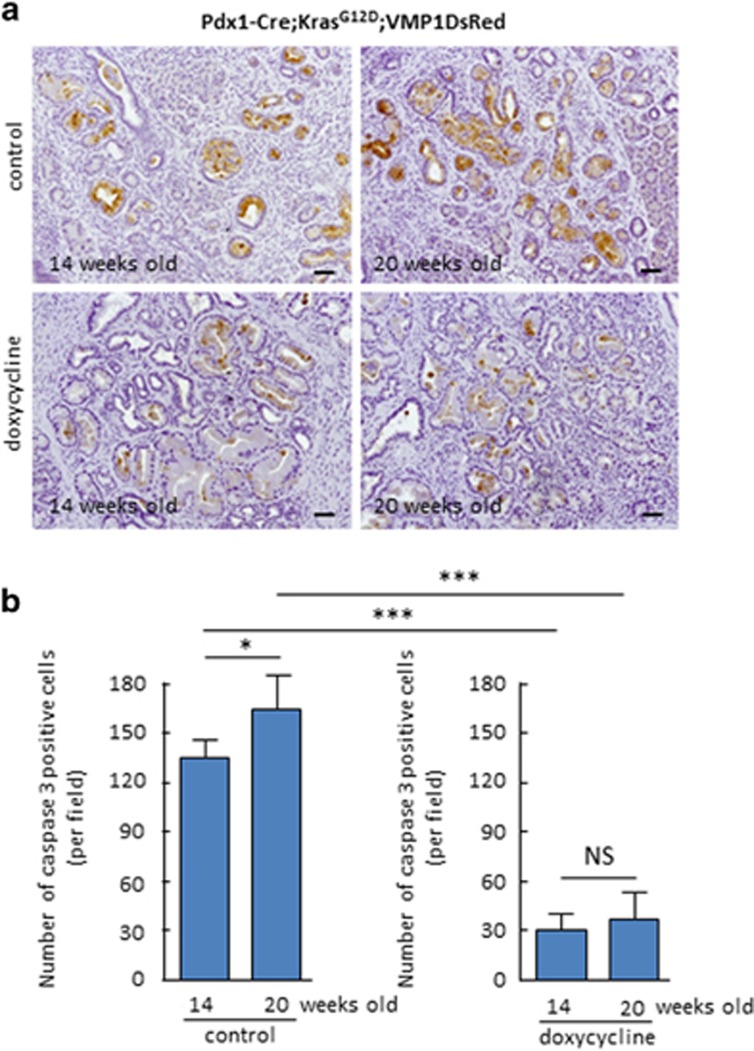
Activity of caspase 3 is decreased in the pancreas of Pdx1-cre;LSL-Kras^G12D^;VMP1DsRed mice treated with doxycycline. Immunostaining with the antibody M30 was performed to estimate the caspase 3 activity in the pancreas of Pdx1-cre;LSL-Kras^G12D^; VMP1DsRed mice treated or not with doxycycline. (**a**) Representative pictures of pancreas from Pdx1-cre; LSL-Kras^G12D^; VMP1DsRed mice stained with the M30 antibody at 14 and 20 weeks old treated with doxycycline dissolved in drinking water. (**b**) Quantification of positive cells per × 20 tissue field in Pdx1-cre;LSL-Kras^G12D^;VMP1DsRed treated with doxycycline or control at 14 and 20 weeks of age. (*P<0.05, **P<0.01, ***P<0.001, and NS corresponds to not significant) mean±S.E.M. Scale bars=50 *μ*m

To define the role of VMP1 in PDCA initiation, we initially determine the levels of PanIN formation in the doxycycline-treated Pdx1-cre;Kras^G12D^;VMP1DsRed mice (Figure 2). As expected control mice sacrificed at 14 and 20 weeks old of age control mice developed extensive ADM as wells as low and high-grade PanINs (average of PanIN per field was 19±5, *n*=6, and 26±6, *n*=5, *P*<0.05). In contrast, animals in which VMP1DsRed expression was induced by doxycycline revealed an increased number of PanINs (average of PanIN per field was 38±10 (*n*=6), and 47±13 (*n*=5, *P*<0.05). Congruent with this result, Figure 3 shows that VMP1DsRed-expressing pancreatic cells exhibited a significant increase in proliferating cells as demonstrated by higher levels of Ki67 positive staining (43±14 *versus* 102±9 at 14 weeks old and 47±9 *versus* 111±12 at 20 weeks old, *P*<0.05). In addition, Figure 4 shows that VMP1DsRed-expressing animals have a decreased in the numbers of apoptotic cells as demonstrated by activated caspase 3 staining (132±11 *versus* 31±8 at 14 weeks old and 159±16 *versus* 37±14 at 20 weeks old, *P*<0.001). As a control we treated Pdx1-cre-Kras^G12D^ mice with doxycycline and found not significant differences in PanIN development. Average of PanIN per field was 21±7 in doxycycline-treated mice (*n*=3), and 18±5 for their control (*n*=3). Combined, these results demonstrate, for the first time, that VMP1 promotes PDAC initiation by oncogenic Kras via a mechanism stimulating cell survival and proliferation.

### VMP1 promotes Kras^G12D^-mediated PDAC initiation through the regulation of autophagy

**Figure 5:**
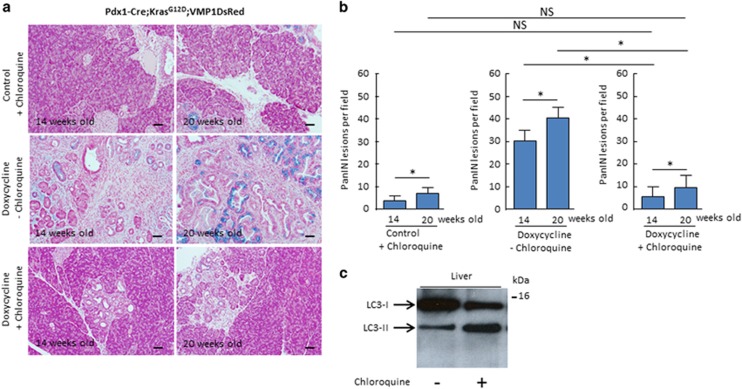
PanINs development is inhibited by the concomitant treatment with chloroquine in Pdx1-cre;LSL-Kras^G12D^;VMP1DsRed mice. (**a**) Representative pictures of pancreas from Pdx1-cre; LSL-Kras^G12D^; VMP1DsRed mice stained with Alcian blue at 14 and 20 weeks old treated with or without doxycycline dissolved in drinking water concomitantly with choroquine or not. (**b**) Number of PanIN lesions per × 20 tissue field in Pdx1-cre; LSL-Kras^G12D^; VMP1DsRed treated with doxycycline or not together or not with chloroquine at 14 and 20 weeks of age. (*P<0.05 and NS corresponds to not significant) mean±S.E.M. Scale bars=50 *μ*m. (**c**) Efficiency of the chloroquine treatment was controlled in liver of these mice by measuring the cleavage of LC3 protein by western blot with an anti LC3 antibody

We next evaluated the effects of inducing the VMP1DsRed protein on autophagy by measuring LC3 cleavage, a well established marker of autophagy. As previously reported by our group and others, the induction of VMP1 promotes cleavage of LC3-II by 7.4±3.1 folds, *P*<0.05 (Figure 1). Congruent with these results, immunohistochemistry (IHC) analysis reveals that the VMP1DsRed signal adopts a punctate distribution pattern, reminiscent to the localization of proteins that mediates cell autophagy (Figure 1). To gain insight as whether the effects of VMP1 in promoting PanIN formation was mediated through its autophagic function, we treated these animals, daily, with chloroquine (60 mg/kg), starting at 3 weeks of age, a period that coincides with a robust initiation of PanIN formation. As a control, we measured the amount of LC3 cleavage in liver. Figure 5 shows that, indeed after this period of treatment, liver cells display a significant accumulation of the LC3-II isoform, revealing that this drug efficiently blocks autophagic flux *in vivo*. We then evaluated the effect of chloroquine on PanIN development in both 14 and 20 weeks old Pdx1-cre; Kras^G12D^;VMP1DsRed mice treated with doxycycline. The results of these experiments are shown in Figure 5. Control animal developed an average of 4.8±1.8, (*n*=4) PanIN lesions at 14 weeks and 7.5±1.9 (*n*=4) at 20 weeks. As expected, the number of PanIN lesions was significantly increased in mice treated with doxycycline (29.8±4.5 (*n*=4) and 41.1±5.5 (*n*=4) at 14 and 29 weeks, respectively, *P*>0.05) but significantly reduced in these animals when treated with chloroquine with an average of 6.2±2.1, (*n*=4), *P*<0.05 and 11.0±5.2, PanINs (*n*=4), *P*<0.05 at 14 and 20 week old, respectively). Thus, we conclude that the effect of VMP1 in promoting PDAC initiation by oncogenic Kras is medicated through its effects on autophagy.

In summary, at the onset of this study, the role of VMP1 in pancreatitis has been established.^15^ However, nothing was known about the effects of VMP1-mediated autophagy on oncogenic Kras-mediated PDAC initiation. Addressing this question became of significant importance since pancreatitis predisposes to cancers but the molecules, which are candidates to mediate this transition, remain a topic of extensive investigation. Our research led to the generation of a new model in which the role of VMP1-mediated autophagy on aspects related to pancreatic carcinogenesis can be reliably study. Using this model, we find that VMP1 exerts a promoting effect on PDAC initiation by mediating the formation of PanIN through an increased in cell proliferation and a decrease in cell death by apoptosis. In addition, we demonstrate that chloroquine, a drug currently being tested in clinical trials for the therapeutics of PDACs, reverse this effect. Thus, combined these results bear both mechanistic and biomedical relevance for better understanding and potentially targeting pathways which are critical for initiating pancreatic carcinogenesis.

## Discussion

Even though the role of pancreatitis as a promoting stimuli for the initiation of PDAC has been extensively documented for both human and mouse, the molecular mediators of this phenomenon remain to be fully characterized. Consequently, the current study was designed to begin filling this gap in knowledge by studying how the pancreatitis-associated protein VMP1, a key regulator of autophagy, cooperates with oncogenic KRAS to give rise to PanINs. This is also important since the role of autophagy in PDAC development remains a controversy. Some studies, for instance, have reported that autophagy promotes cancer development whereas others have demonstrated the opposite effect. Our experimental design involved the development of a new mouse model in which the oncogene Kras was activated in the pancreas by the expression of the Pdx1-cre while VMP1 was simultaneously induced by doxycycline. We observed that expression of the VMP1 is accompanied by a significant increase in the number of PanIN lesions and the concomitant treatment with chloroquine abolishes this effect. We concluded that the promoting effect of VMP1 on KRAS-induced PDAC initiation is mediated through its role as a regulator of autophagy. Thus, this information extends the repertoire of pancreatitis-associated proteins as well as autophagy regulators, which can contribute to pancreatic carcinogenesis.^16, 17, 18^

In light of these results it becomes important to review and discuss the known functions of VMP1. For instance, it has been established that this protein is involved in the initiation of autophagy since, cells lacking VMP1 have elevated and aberrant PtdIns3P signaling on the ER, resulting in an increased and persistent recruitment of Atg18 and other autophagic proteins.^14^ In addition, although ULK1 and ATG5 are separated in the genetic hierarchy, these proteins synchronously accumulate at preexisting VMP1-positive punctate structures, followed by recruitment of ATG14, ZFYVE1, and WIPI1.^19^ Moreover, VMP1 bind directly to the BH3 motif of beclin 1 leading to the formation of a complex with the Class III phosphatidylinositol-3 kinase hVps34, a key positive regulator of autophagy, at the site where autophagosomes are generated. Interestingly, the interaction between beclin 1 and VMP1 leads to the dissociation of Bcl-2 from beclin 1 increasing the intracellular amount of the available beclin 1 to induce autophagy.^20^ Furthermore, VMP1 regulates autophagosome formation by shortening the duration of omegasomes and therefore accelerating the autophagy flux.^13^ Lastly, in Dictyostelium, VMP1-inactivated mutant cells showed accumulation of massive ubiquitin-positive protein aggregates containing the autophagy marker Atg8 and p62 homologue demonstrating that VMP1 is required for the clearance of these ubiquitinated protein aggregates through autophagy.^21^ Thus, VMP1 is both, a regulator of autophagy during pancreatitis since its overexpression activates signaling to promote autophagosome formation and also a transmembrane protein integrated into the autophagosome. However, until the current study was completed nothing was known about the role of VMP1-mediated autophagy and pancreatic carcinogenesis. This observation is of significant importance since the role of autophagy in cancer development is multiple and complex. In fact, a plethora of studies indicate that autophagy may either favor or constrain tumor development and progression depending of the intracellular pathways considered. For this reason, it is difficult to predict as whether a particular regulator of autophagy would have a positive or negative effect on neoplastic transformation making the type of experimentation reported in this study a ‘sine qua non' effort.

An important question in the field of pancreatology is how pancreatitis promotes the formation of PanINs.^22^ An insight into this question is provided by studies, which show that during pancreatitis autophagy is systematically activated, frequently as a protective function, to avoid the development or help in the recovery phase of the disease.^23, 24^ It is likely that, at least part of the protective effect of autophagy is related to both its role in the sequestration of zymogen granules that contain the deleterious enzymes that kill acinar cells and by supporting the enhanced metabolic rate that accompany cell growth during regeneration.^15^ We therefore hypothesized that, in the presence of oncogenically active KRAS, autophagy as induced by pancreatitis-associated proteins such as VMP1 may serve at least as one of the molecules that link this disease with the process of cancer initiation. In fact, the data described as result of the current study support this idea.

It is also important to discuss the role that autophagy plays in cancer development, as it appears to be varied and complex. In fact, it has been previously demonstrated that autophagy mediates the mitotic senescence transition.^25^ Autophagy is activated upon acute induction of this cellular process to facilitate the senescence associated secretory phenotype, which also creates an inflammatory microenvironment.^26^ Senescence is known to be a major anticancer pathway that is activated in response to the oncogenic Kras activation and, in this context therefore, senescence enhanced by autophagy could help to inhibit the oncogenic effect induced by Kras^G12D^. In addition, autophagy activation acts as an antiapoptotic factor is some tissues, depending of the biological circumstances.^27, 28^ Moreover, following its oncogenic activation, Kras induces a metabolic stress due to the increased metabolic needs of the cell that can be bypassed by activating the autophagy as a source of energy.^29, 30^ All these above mentioned mechanisms may modulate, favor, or even antagonize tumor development and progression depending of the intracellular pathways, which is operational at a particular state of the cell carrying Kras mutations, and may likely explain the often contrasting results previously reported on the role of autophagy in cancer.

Similarly important is to consider that autophagy is suspected to be a mediator of cancer resistance to both radiotherapy and chemotherapy with certain drugs.^31, 32^ However, the mechanisms by which autophagy acts in cancer resistance is also complex since anticancer treatments often damage intracellular organelles thereby creating protein aggregates that must be cleared by autophagy is to protect cells. On the other hand, autophagy has also been reported to act as a mediator of chemotherapy-induced cell death in cancer.^33^ The mechanism of action of the autophagy-dependent cell death is not clearly established although it seems to be mediated throughout the caspase 3 activation.^33^ Therefore, just as we discussed for the role of autophagy in cancer initiation and progression, reports supporting or contradicting its function in chemotherapeutics are abundant in the literature.^34, 35^ Nevertheless, the fact that cancer treatment induces autophagy systematically is currently unambiguous.^36^ Thus, congruent with this knowledge, co-treatment with the autophagy inhibitor chloroquine improves the effect of a significant number of anticancer drugs.^37, 38, 39, 40, 41^

In conclusion, we report that VMP1 promotes PanIN development in the presence of an oncogenic Kras signaling pathway activation through its role in autophagy.

## Materials and Methods

### Mice

We developed a DNA construct named Ptight-VMP1DsRed-IRES-Luciferase Renilla-SV40 polyA by subcloning the cDNA encoding VMP1-DsRed fusion proteins into the EcoRI/NheI restriction sites and the cDNA encoding luciferase renilla into the BamHI/EcoRV restriction sites of the pTRE-Dual1 plasmid (Clontech Laboratories Inc., Palo Alto, CA, USA). The construct was sequenced and its response to the doxycycline evaluated on HEK293T cells. The transgene was separated from plasmid vector sequences by XhoI digestion and purified after agarose gel electrophoresis with Elutip-d columns (Schleicher & Schuell, Dassel, Germany) and ethanol precipitation. Microinjections into fertilized oocytes derived from B6D2 mice were realized at the Service d'Expérimentation Animale et Transgenèse (SEAT, CNRS, Villejuif, France). Founders were identified by polymerase chain reaction (PCR) analyses of tail DNAs from 2-week-old mice using oligonucleotides specific to the DsRed-GTFw (5′-CTTCAAGACCGTGTACAAGGCCAAG-3′) and IRES-GTRev (5′-GGGTCGCTACAGACGTTGTTTGTCTTC-3′) and RlucFw (5′-TTTTTGGATCCATGACCAGCAAGGTGTACGACCC-3′) and RlucRev (5′-TTTTGATATCCTGCTCGTTCTTCAGCACTCTCTCCACG-3′). PCR conditions are available upon request.

*Pdx1*-cre and *LSL*-Kras^G12D^ mice strains have been described elsewhere.^17, 42^ Because animals are from different genetic backgrounds, we systematically used littermate control and experimental mice. Mice were kept within the Experimental Animal House of the Centre de Cancérologie de Marseille (CRCM), pole Luminy, following institutional guidelines.

Doxyclycline Hyclate and Hydroxychloroquine were purchased from Sigma-Aldrich. Transgenic mice were exposed to doxycycline (250 mg/l) dissolved in 5% sucrose supplied as drinking water, which was protected from the light and exchanged every 3 days. Mice received daily intraperitoneal injections of chloroquine (60 mg/kg), or saline, started at the end of 3 weeks old and for 10 or 16 weeks.

### Histology and IHC

Pancreatic sections were fixed in 4% paraformaldehyde and paraffin embedded. H&E and Alcian blue staining were performed using standard procedures. Sections were probed with primary antibodies against Ki67 (BioLegend, San Diego, CA) to estimate the proliferation activity, against LC3 (Medical & Biological Laboratories, Nagoya, Japan) to measure autophagosome formation and against the cleaved CK18 to study the apoptotic activity. In fact, during apoptosis, CK18 undergoes dramatic reorganization and is cleaved by the caspases, generating an apoptosis-specific neo-epitope recognized by the monoclonal antibody M30 (Cell Signaling, Beverly, MA). Samples were examined in an Eclipse 90i Nikon microscope.

### Quantification of lesions per mouse

Number of lesions per field was counted and the types of lesion were classification on H&E-stained slides. Quantification represents the average of 15–20 × 20 fields of view from 4 to 6 mice of each genotype.

### Immunoblotting

Protein extraction was performed on ice using total protein extraction buffer: 50 mM HEPES pH 7.5, 150 mM NaCl, 20% SDS, 1 mM EDTA, 1 mM EGTA, 10% glycerol, 1% Triton, 25 mM NaF, 10 *μ*M ZnCl_2_, 50 mM DTT. Before lysis, protease inhibitor cocktail at 1:200 (Sigma-Aldrich, NUPR1340), 500 *μ*M PMSF, 1 mM sodium orthovanadate, and 1 mM *β* glycerophosphate were added. Protein concentration was measured using a bicinchoninic acid assay protein assay kit (Pierce Biotechnology). Protein samples (60 *μ*g) were denatured at 95 °C and subsequently separated by SDS-PAGE gel electrophoresis. After transfer to nitrocellulose, membrane was blocking with 1% BSA, samples were probed with primary antibody followed by a horseradish peroxidase couple secondary antibody. Primary antibodies against LC3 was from Medical & Biological Laboratories (Nagoya, Japan) and antibody against VMP1 was homemade.^11^ Polyclonal antibody against *β*-Tubulin was used as a loading control was from Sigma-Aldrich (St. Louis, MO). Image acquisition was made in Fusion FX image acquisition system (Vilber Lourmat) and bands were quantified ImageJ software (NIH).

### RT-qPCR

One μg RNA from pancreas was reversed transcribed using the Go Script reagent (Promega) according to manufacturer's instructions. Real-time quantitative PCR for VMP1 was performed in a Stratagene cycler using Takara reagents with the following primers sequences 5′-CTGGCAGTTCAAAAACTAGTAC-3′ and 5′-CCGGGGACAGCACCAATGAAAG-3′ which recognize both human and mouse transcripts.

### Statistical analysis

One-way variance analysis was used to calculate significances between Pdx1-Cre;Kras^G12D^;VMP1DsRed and control per tissue (%) as previously described.^43^ To compare KI67 and caspase 3 activity one-way variance analysis was used, *P* value was calculated used delta Ct as described by Yuan *et al.*^44^ For compared numbers of lesion per field in between groups at 14 and 20 weeks old, a two-way variance analysis was used. Values were expressed as mean±S.E.M. All tests of significance were two-tailed and the level of significance was set at 0.05. All statistics test were performed using IBM SPSS statistics 21.

## Abbreviations

VMP1: vacuole membrane protein 1
PDAC: pancreatic ductal adenocarcinoma
PanIN: pancreatic intraepithelial neoplasia
GFP: green fluorescent protein
DsRed: red fluorescent protein
GEMM: genetically engineered mouse model
IHC: immunohistochemistry
PCR: polymerase chain reaction
HEPES: 4-(2-hydroxyethyl)-1-piperazineethanesulfonic acid
EDTA: ethylenediaminetetraacetic acid
EGTA: ethylene glycol tetraacetic acid
DTT: 1,4-dithiothreitol
BCA: bicinchoninic acid assay
