# Supplementary figures and images for: The pancreatitis-associated protein VMP1, a key regulator of inducible autophagy, promotes KrasG12D-mediated pancreatic cancer initiation
Source: Cell Death Dis. 2016 Jul 14;7(7):e2295–. doi: 10.1038/cddis.2016.202 (PMC4973346; doi:10.1038/cddis.2016.202)

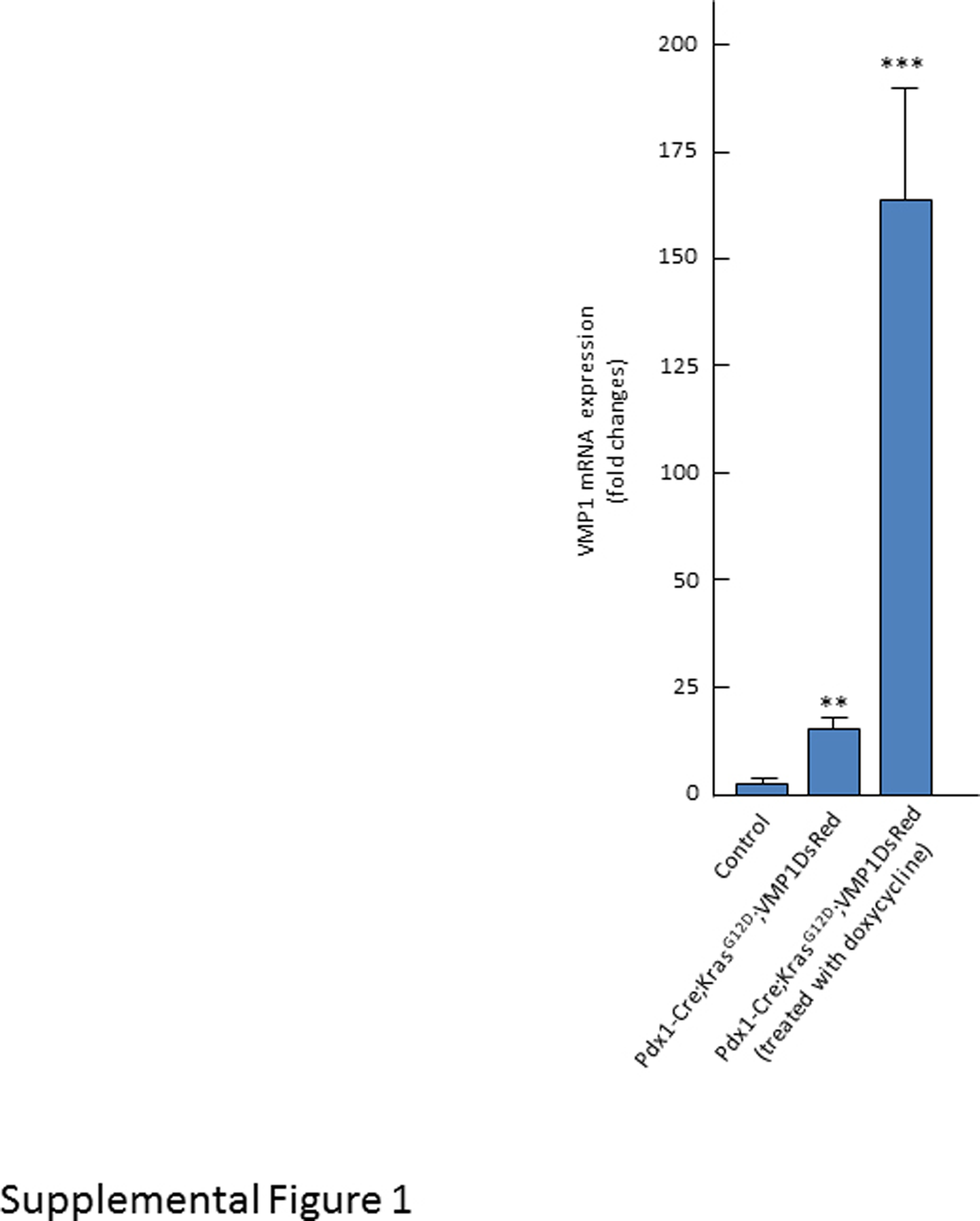

Supplement: Supplementary Figure 1 [file cddis2016202x1.tif]
