# Supplementary material for: The pancreatitis-associated protein VMP1, a key regulator of inducible autophagy, promotes KrasG12D-mediated pancreatic cancer initiation
Source: Cell Death Dis. 2016 Jul 14;7(7):e2295–. doi: 10.1038/cddis.2016.202 (PMC4973346; doi:10.1038/cddis.2016.202)
Supplement: Supplementary Information [file cddis2016202x2.doc]

**Supplemental Figure 1**

RNA from pancreases was reverse transcribed using the Go Script reagent. qRT-PCR for VMP1 was performed in a Stratagene cycler using primers sequences which recognizing both human and mouse transcripts. ** correspond to p>0.01 and *** to p>0.001.) mean ± S.E.M. (n=3).
